# Conversion of recombinant human ferritin light chain inclusion bodies into uniform nanoparticles in *Escherichia coli* for facile production

**DOI:** 10.1002/elsc.202100164

**Published:** 2022-04-23

**Authors:** Xiaotong Song, Yongxiang Zheng, Yongdong Liu, Huan Meng, Rong Yu, Chun Zhang

**Affiliations:** ^1^ Department of Biopharmaceutics Key Laboratory of Drug‐Targeting and Drug Delivery System of the Education Ministry Sichuan Engineering Laboratory for Plant‐Sourced Drug and Sichuan Research Center for Drug Precision Industrial Technology West China School of Pharmacy Sichuan University Chengdu P. R. China; ^2^ State Key Laboratory of Biochemical Engineering Institute of Process Engineering Chinese Academy of Sciences Beijing Beijing P. R. China

**Keywords:** Capto Butyl, *E. coli* soluble expression, pBV220 plasmid, precipitation procedure, recombinant human ferritin light chain

## Abstract

Prokaryotic expression systems are widely used to produce many types of biologics because of their extreme conveniences and unmatchable cost. However, production of recombinant human ferritin light chain (rhFTL) protein is largely restrained because its expression in *Escherichia coli* tends to form inclusion bodies (IBs). In this study, a prokaryotic expression vector (FTL‐pBV220) harboring the rhFTL gene was constructed using a pBV220 plasmid. The tag‐free rhFTL was highly expressed and almost entirely converted to soluble form, and thus the rhFTL was successfully self‐assembled into uniform nanoparticles in *E. coli*. To establish a simplified downstream process, a precipitation procedure based on the optimized incubation temperature, pH condition, and ionic strength was developed to remove impurities from the crude lysate supernatant. The rhFTL retained in the clarified supernatant was subsequently purified in a single step using Capto Butyl column resulting in a considerable recovery and high purity. The purified rhFTL was characterized and verified by mass spectrometry and spectral and morphological analyses. The results revealed that rhFTL exhibited highly ordered and fairly compact structures and the spherical structures were preserved.

**Abbreviations**
CD: ircular dichroism
DLS: dynamic light scattering
DSC: differential scanning calorimetry
ESI‐MS: electrospray ionization mass spectrometers
rhFTL: recombinant human ferritin light chain
RP‐HPLC: reversed‐phase high performance liquid chromatography
TEM: transmission electron microscopy

## 1 INTRODUCTION

Ferritin is the major iron‐storage protein existing in almost all living systems and it is mainly responsible for iron storage, iron release, and anti‐oxidation [1, 2]. Vertebrate ferritins are typically composed of two types of subunits: ferritin heavy chain (FTH) and ferritin light chain (FTL), whereas plant and bacterial ferritins only contain heavy chains [1]. Naturally occurring human ferritin has a hollow spherical shape and generally contains 24 subunits which are comprised of different ratios of heavy to light chain. The ratio of FTH and FTL varies in depending on the tissue distribution and pathological changes [3, 4]. Generally, human ferritin has an external diameter of about 12 nm and an interior cavity of about 8 nm. The two subunits of human ferritin share approximately 53% sequence homology and exist in extremely similar advanced structures as a four‐helix bundle. Importantly, there is a higher sequence homology between the same isoforms from different species than that between FTH and FTL within the same species.

Although FTH and FTL share over half of the amino acid sequence homology and exhibit extremely similar structural profiles, the two chains play distinct roles in the process of iron storage [5, 6]. FTH contains a catalytic ferroxidase site that catalyzes the oxidation of Fe (II) to Fe (III). In contrast, FTL provides an effective nucleation site for iron, which accelerates the formation and demineralization of iron nuclei. The electrons released from FTH during iron‐oxidation were transported across the ferritin cage specifically through the L‐chains and the inverted electron transport through the L‐chains also accelerated the demineralization of ferritin [7]. FTL recently was found to be capable of binding to scavenger receptor class A member 5 (SCARA5) which is mainly expressed on the surface of kidney podocytes and splenic littoral endothelial cells and endows the human natural ferritin with an alternative non‐transferrin iron delivery pathway [8, 9, 10]. To date, research efforts concerning ferritin remain focus on its functional mechanisms and regulation. Besides, FTH and FTL also are frequently used as a protein‐based nanoparticle carrier to present peptide‐based epitopes for vaccine development using chemical crosslinking because of its extreme stability and safety [11]. Thus, establishment of facile strategies for the preparation of FTH and FTL is of substantial importance.

**PRACTICAL APPLICATION**

*Escherichia coli* expression systems are frequently used to produce recombinant proteins. However, heterologous proteins always suffer the risk of the formation of inclusion bodies (IBs), in which the proteins aggregate into an insoluble form during synthesis in *E. coli*. In this article, the tag‐free rhFTL was expressed in the form of IBs in *E. coli* when using a pET‐30a(+) vector as previously reported, and it was successfully converted to soluble form using a pBV220 vector and thus achieved self‐assembly into uniform nanoparticles in the cytoplasm of *E. coli*. We deduced that the retarded FTL expression rate and the potential folding chaperones resulting from the induction manner of pBV220 vector system or other heat‐shock response in host probably attribute to this conversion. The main outcome of our work is that pBV220 vector can potentially be used for soluble expression of those proteins that tend to form inclusion body when expressed in *E. coli*.

Although the extraction of natural ferritin from blood and organ tissues and preparation of recombinant FTH nanoparticles using prokaryotic bacteria were achieved, the downstream processes were difficult to operate [12, 13, 14, 15]. Moreover, in contrast to the heavy chain, the production of recombinant FLC using prokaryotic expression system was largely restrained owing to its higher hydrophobicity, which resulted in the formation of IBs during expression in *E. coli* [14, 16]. Besides, to our knowledge, very few studies have focused on developing a facile downstream bioprocess for the production of rhFTL in *E. coli*. Benefiting from its fast growth rate, considerable expression yield, and extremely inexpensive culture medium, *E. coli* expression system is widely adopted to produce many types of biologics, such as enzymes, cytokines, and subunit vaccines. [17]. However, development of downstream process is a challenge for *E. coli*‐expressed recombinant proteins, particularly those which are synthesized in bacteria in a soluble form, due to the presence of substantial amount of impurities resulting from the host cell lysate [18].

Herein, a prokaryotic expression vector harboring the recombinant human FLC gene (rhFTL‐pBV220) was constructed using a pBV220 plasmid that contains a temperature‐sensitive pR/pL promoter known to efficiently drive gene expression at 42°C. Tag‐free rhFTL was highly expressed and importantly, was converted to a soluble form. Thus it was successfully self‐assembled into uniform nanoparticles in *E. coli*. To establish a simplified downstream process, a non‐chromatographic precipitation procedure based on incubation temperature, pH value, and sodium chloride concentration, was studied and optimized. As a result, the purity of rhFTL could reach 98% when it was achieved from the crude lysate by only one step of hydrophobic interaction chromatography. The purified rhFTL was characterized and verified by mass spectrometry, and spectral and morphological analyses. In addition, the thermal stress response profile was analyzed to interpret the behavior of rhFTL in the precipitation process.

## 2 MATERIALS AND METHODS

### 2.1 Materials

Yeast extract and tryptone were purchased from Oxoid and the other reagents were of analytical grade. Pre‐packed Capto Butyl column (Hiscreen, 1 × 4.7 mL) was purchased from Cytiva (USA). T‐100 PCR from BioRad (USA), T&J‐A type 10‐L fermentor (T&J Bio‐engineering, China), AH‐NANO (ATS, China), ÄKTA pure (GE healthcare, USA), HPLC Agilent 1100 (USA), *Proteonavi C4* column (Shiseido, Japan), *TSKgel G3000SWxl* (TOSOH, Japan), Zetasizer Nano ZS90 (Malvern Panalytical, UK), H‐600 Transmission electron microscope (Hitachi, Japan), Differential scanning calorimetry (GE Healthcare, USA), J‐810 circular dichroism spectrometer (Jasco, Japan), RF‐6000 spectro fluorophotometer (Shimadzu, Japan), and Dynamic light scattering (Malvern Panalytical, UK).

### 2.2 Construction of FTL‐pBV220 expression vector

To achieve a higher expression level, the FTL‐pET‐30a(+) plasmid containing the human FTL (UniProtKB ‐ P02792) gene and the DNA sequence codon of FTL was optimized to adapt to *E. coli* expression system. Two strands of primers, GTTAAAAATTAAGGAGGAATTCCATATGAGCAGCCAGATTCGCC and GGTCGACGGATCCTTAATCATGTTTCAGGGTCAGG, were synthesized by Shanghai Shenggong Biological Co., Ltd. The plasmid extraction kit was used to extract FTL‐pET‐30a(+) plasmid. *Nde*Ⅰ restriction site was introduced in the front of FTL target gene using a forward primer. Subsequently, PCR was performed to obtain a pBV220 linear fragment without multiple cloning sites (MCS) between *Nde*I and *Bam*HI. The target gene was inserted between the *Nde*I and *Bam*HI restriction sites by one‐step cloning to construct FTL‐pBV220 plasmid. The FTL‐pBV220 plasmid subsequently was transformed into *E. coli* BL21 (DE3) competent cells according to the instructions. The positive clone was screened on a solid culture medium plate with 50 μg/mL ampicillin after 36 h incubation at 30°C.

### 2.3 Fermentation

The *E. coli* BL21 (DE3) strain harboring the FTL‐pBV220 plasmid was firstly incubated in 150‐mL *Luria‐Bertani* (LB) medium supplemented with 50 μg/mL of ampicillin and was subsequently incubated at 30°C overnight. The bacterial culture was then transferred into 3 L of 2 × LB medium containing 0.5% glycerol and 100 μg/mL of ampicillin in a 10‐L fermenter system. The culture temperature was maintained at 30℃ and the parameters were adjusted to maintain dissolved oxygen (DO) no less than 20%. When the bacteria density (OD_600_) reached approximately 5.0, the culture temperature was changed to 42℃ to drive FTL gene expression for another 4 h. Fed‐batch operation was started after the temperature reached 42℃ with a continuous addition speed of 150 mL/h (10× LB medium supplemented with 5.0% glycerol). After that, the bacteria were harvested by centrifuge at 6000 rpm for 15 min. The collected bacteria‐pellets were re‐suspended in buffer (10 mM PB, 1.0 mM EDTA pH 7.0) with a proportion of approximately 15% (w/v) and disintegrated by AH‐NANO high‐pressure homogenizer (ATS, China) for five cycles under the pressure of 700–800 bar. The crude lysate supernatant was obtained by centrifugation at 10,000 rpm at 4°C for 30 min stored at –20°C until use.

### 2.4 Precipitation process optimization

During this procedure, the protein recovery and purity of FTL protein in different conditions varying the heat treatment temperature (70°C or 75°C), solution pH (4.0, 4.4, and 4.8), and concentration of NaCl (0, 0.5, 1.0, 1.5, and 2.0 M) was evaluated. The crude lysate supernatant (500 μL) was mixed with 500 μL precipitation buffer (200 mM NaAc/AcH) at different pH and NaCl concentrations (1.0 mL in each 1.5 mL tube) to adjust to the expectant pH conditions and ionic strengths. The samples were water incubated at 70°C or 75°C for 10 min, and then were immediately chilled in ice‐water bath for 10 min. After that, the samples were then clarified by centrifugation at 12,000 rpm for 30 min. The concentration of residual proteins in the supernatant after precipitation treatment was determined by a modified BCA method (BCA protein assay kit, Sangon Biotech). The content of the target protein in the supernatant and precipitates were analyzed by SDS‐PAGE (13.5%) and roughly estimated by ImageJ software.

### 2.5 Purification of rhFTL by Capto Butyl

The crude lysate supernatant was mixed with the optimized buffer (200 mM NaAc/AcH, 2.0 M NaCl pH 4.4) in a ratio of 1:1 and divided into 15‐mL centrifuge tubes, each filled with 10 mL. The tubes were placed in a water bath at 75℃ for 10 min and chilled in ice water. The mixture was subsequently clarified by centrifugation at 10,000 rpm for 30 min and the supernatant was discreetly drawn out. The solution conductivity was modulated to about 160 mS/cm by 4 M (NH_4_)_2_SO_4_ and the solution pH was adjusted to pH 6.5 by 1.0 M Tris. The clarified supernatant then proceeded to purification system (AKTA pure, GE healthcare) and a pre‐packed Capto Butyl column was implemented and equilibrated with buffer A (10 mM PB, 1.2 M (NH_4_)_2_SO_4_ pH 6.5). After loading sample, the column was washed with 30% buffer B (10 mM PB pH 6.5) until the UV280 signal baseline tended to be stable. Eventually, the protein was gradually eluted by 90% buffer B. All the intermediate samples were collected during the whole process and eventually analyzed by SDS‐PAGE (13.5%). The resulted rhFTL was dialyzed to PBS buffer and finally stored at 4℃.

### 2.6 Sodium dodecyl sulfate polyacrylamide gels electrophoresis (SDS‐PAGE)

SDS‐PAGE (13.5%) analysis was performed as described by *Laemmli* [19]. The samples mixed with reducing loading buffer were denatured at temperatures above 95°C for 5 min. Electrophoresis was initially performed at 80 V for 30 min and then conducted at 160 V until bromophenol blue reached the bottom of the gels. The gel was stained with CBB R‐250 dye for 30 min and then decolorized to obtain a clear background.

### 2.7 Reversed‐phase high performance liquid chromatography (RP‐HPLC)

An Agilent 1100 system was employed to analyze the purity of the rhFTL protein. The *Proteonavi C4* column (4.6 × 250 mm, Shiseido) was pre‐equilibrated by 60% solution A (ddH_2_O, 0.1% TFA) and 40% solution B (CH_3_CN, 0.1% TFA) with a constant flow rate of 0.5 mL/min. The samples were loaded onto the column using an auto‐sampler. The chromatographic conditions were as follows: 0–5 min, solution gradient was linearly increased from 40% to 45% solution B; 5–30 min, further linear from 45% to 65% solution B; 30–45 min, regeneration, 100% solution B. The elution was measured using a diode array detector and the signal was recorded at a wavelength of 280 nm.

### 2.8 HPLC‐ESI‐MS

LCQ DecaXP mass spectrometer connected to an outlet of the Agilent 1100 system was employed to determine the detailed molecular mass of rhFTL protein. The sample was injected into a ZORBAX 300SB‐C18 column (5.0 μm, 4.6 × 250 mm) using an auto‐sampler. The mobile phase A was ddH_2_O supplemented with 0.1% formic acid and the mobile phase B was acetonitrile supplemented with 0.1% formic acid. The system was pre‐equilibrated by 5% mobile phase B. The chromatographic conditions: injected sample volume, 20 μL; flow rate, constant at 0.2 mL/min; 0–20 min, the mobile phase B gradient was linearly increased from 5% to 90%; 20–70 min, 90% mobile phase B. The UV signal was recorded at a wavelength of 215 nm. The HPLC system pipe outlet was connected to the mass spectrometer. The spray voltage was set to 4.5 kV, the capillary temperature was maintained at 275℃ and the scan m/z range was set from 300 to 2000.

### 2.9 Circular dichroism (CD)

Far‐UV CD analysis of rhFTL was performed using a J‐810 spectrometer (Jasco, Japan) at room temperature. The path length of the quartz cuvette was 1.0 mm and the protein concentration was ∼0.3 mg/mL in PBS buffer. The scan wavelength ranged from 260 to 190 nm. The scanning rate was 1000 nm/min with a response of 0.1 s and the wavelength bandwidth was 1.0 nm. Each sample was measured five times, and the final spectrogram indicated the average. The resulted data were processed by deducting the blank buffer signal.

### 2.10 Fluorescence spectroscopy

RF‐6000 fluorescence spectrophotometer (Shimadzu, Japan) was employed to investigate the intrinsic fluorescence emission profile of rhFTL in PBS buffer. The protein sample was diluted to about 0.1 mg/mL and the path length of the cuvette was 1.0 cm. The excitation wavelength was constant at 280 nm, and fluorescence emission signals were recorded from 260 to 450 nm.

### 2.11 High performance size exclusion chromatography (HP‐SEC)

HP‐SEC analysis was performed on an Agilent 1100 system using a *TSKgel G3000SWxl* column (TOSOH, Japan). The elution buffer was PBS containing 0.1 M Na_2_SO_4,_ and the flow rate was set at 0.5 mL/min. The samples were loaded onto the column by an auto‐sampler and the elution was measured using a diode array detector. The signal was recorded at a wavelength of 280 nm.

### 2.12 Dynamic light scattering (DLS)

The rhFTL particle size was determined using a Zetasizer Nano ZS90 instrument (Malvern, UK). Samples were clarified by centrifugation at 12,000 rpm for 15 min and further filtered using 0.2‐μm filters at room temperature. Each sample was measured five times and the final data represented the average.

### 2.13 Transmission electron microscopy (TEM)

H‐600 transmission electron microscope was used to observe the morphological features of rhFTL. The sample concentration was approximately 1.5 mg/mL and it was dropped onto a glow discharged and carbon‐coated electron microscopy grid. After fixation for 1 min at room temperature, the excess sample solution was removed using a filter paper. Subsequently, the grid was cleaned with double‐distilled water and negatively stained with 2% uranyl acetate for 45 s. Excess dyes were cleaned, and the grid was air‐dried prior to observation.

### 2.14 Differential scanning calorimetry (DSC)

The thermal response profile of rhFTL was analyzed using micro‐VP DSC (GE Health, USA). The rhFTL sample solution was exchanged to PBS buffer, and the protein concentration was diluted to approximately 1.0 mg/mL. The measurement was obtained by linearly increasing the incubation temperature from room temperature to 110℃ at a speed of 1.0℃/min. The sample curve was processed by subtracting the blank buffer curve.

### 2.15 Protein concentration determination

Protein concentration, if not otherwise specified, was determined by the Bradford method and bovine serum albumin (BSA) was used as a reference.

## 3 RESULTS

### 3.1 Bacteria fermentation

**FIGURE 1:**
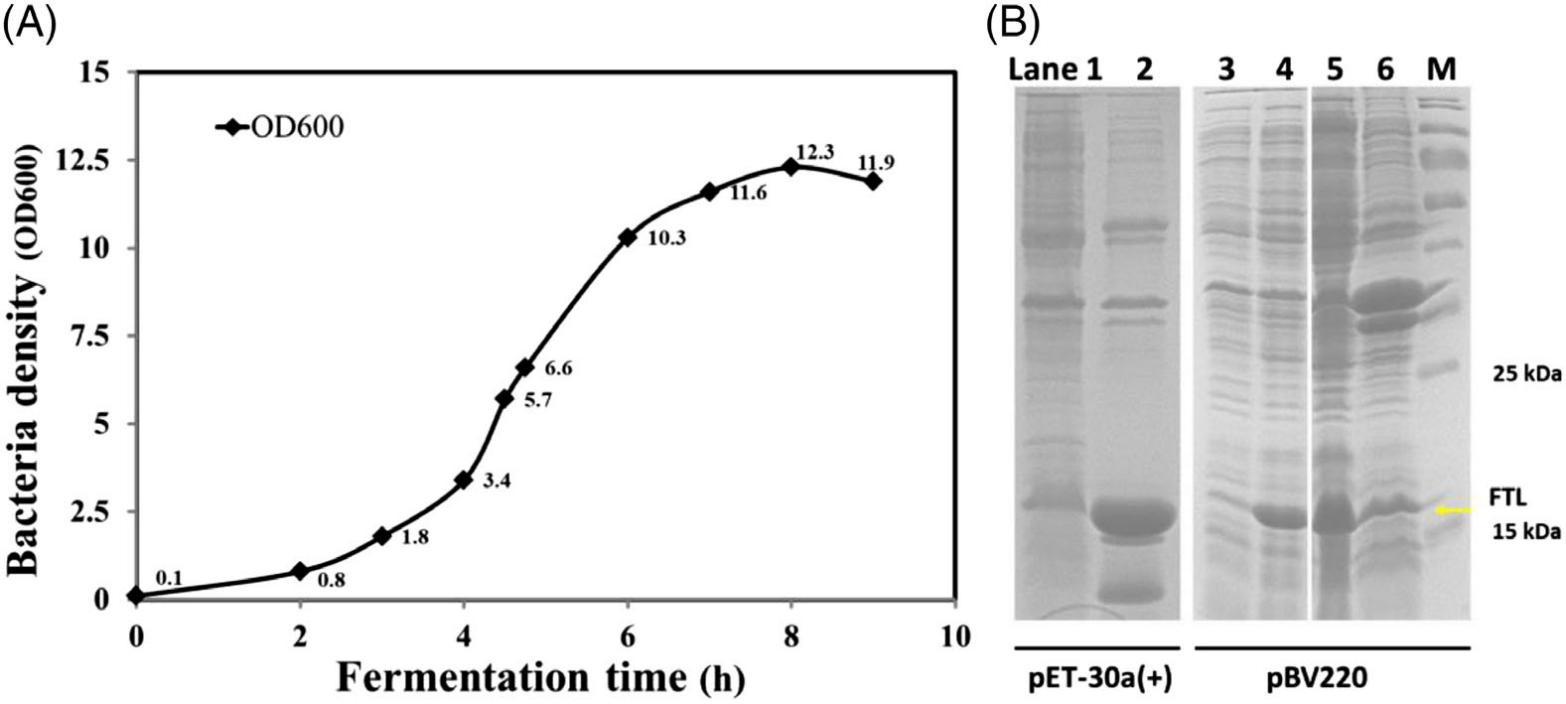
(A) The growth curve during fed‐batch fermentation. (B) SDS‐PAGE analysis of the expression of rhFTL in *E. coli*. Lane 1–2, rhFTL expression by pET‐30a(+) vector (culture at 37℃ in LB, induction by 1.0 mM IPTG for 4 h). lane 1, crude lysate supernatant; lane 2, crude lysate precipitates; lane 3–6, rhFTL expression by pBV220 vector (culture at 30℃ in LB, induction by 42℃ for 4 h); lane 3, pre‐induction; lane 4, 4.0 h post‐induction; lane 5, crude lysate supernatant; 6, crude lysate precipitates

The *E. coli* bacteria growth curve is shown in Figure 1A and about 72.0 g wet cells were finally obtained after fed‐batch fermentation. According to the SDS‐PAGE results (Figure 1B), rhFTL was expressed entirely in the form of IBs in the pET‐30a(+) vector as previously reported [14, 15]. By using pBV220 plasmid, the expression of rhFTL was successfully and effectively converted to its soluble form. The rhFTL was highly expressed in *E. coli* and the expression level of FTL was approximately 20–30% in total protein, as roughly estimated by band scanning.

### 3.2 Precipitation process optimization

**FIGURE 2:**
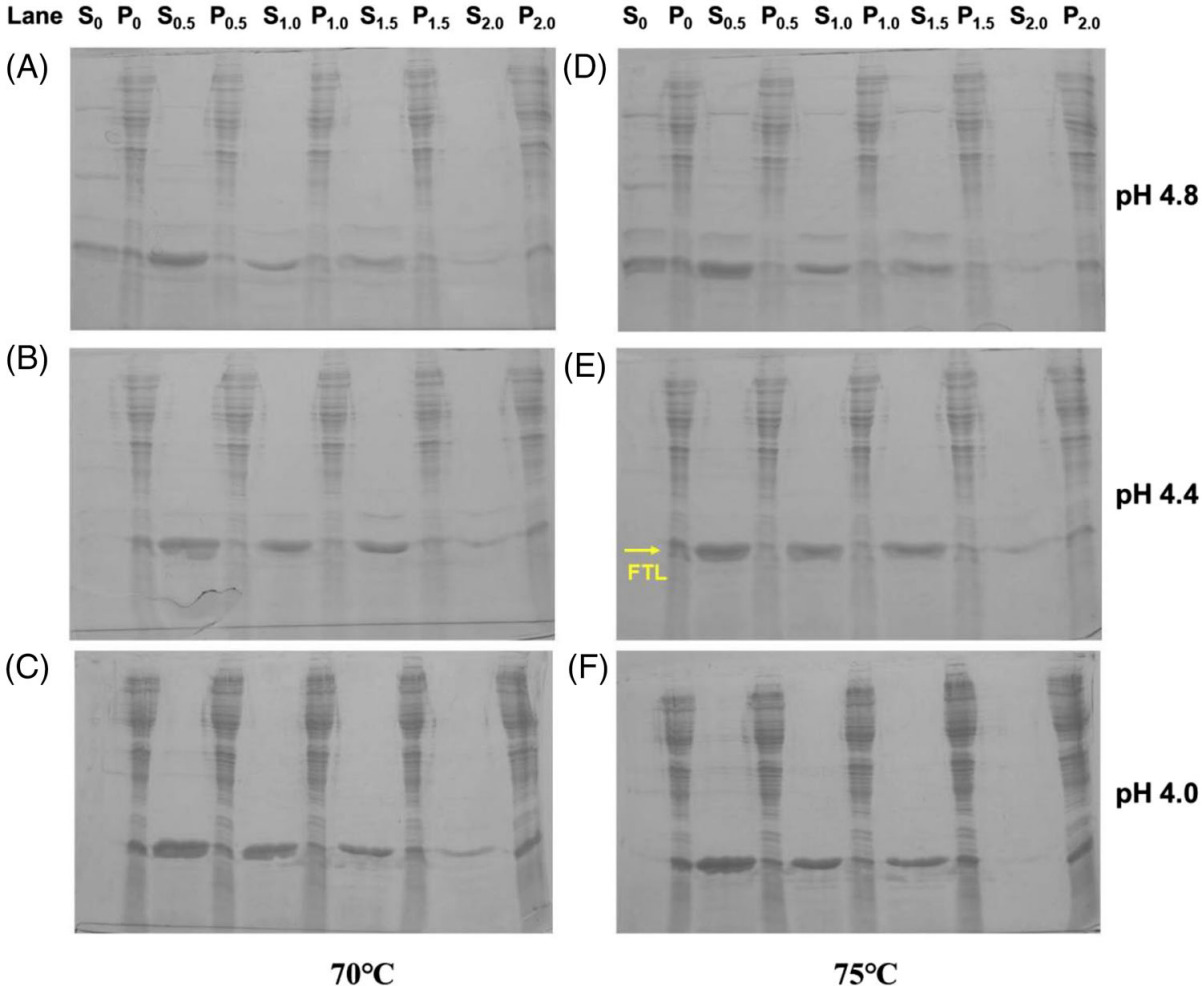
Precipitation process optimization (SDS‐PAGE). (A) pH 4.8, 70℃; (B) pH 4.4, 70℃; (C) pH 4.0, 70℃; (D) pH 4.8, 75℃; (E) pH 4.4, 75℃; (F) pH 4.0, 75℃. Abbreviations: S, clarified supernatant; P, precipitates; subscript no. represents sodium chloride concentration

The outcomes of the precipitation process optimization are summarized in Table 1 and the SDS‐PAGE analysis of the samples is shown in Figure 2. According to the results, most of the unwanted proteins could be efficiently precipitated at 70°C and 75°C, but a few protein bands, especially in the high molecular weight range, remained clearly visible when the solution pH was at 4.8 or above. The whole recovery of the retained proteins in the supernatant notably decreased when the solution pH was reduced. At pH 4.4 and pH 4.0 without the presence of NaCl, the proteins were almost completely precipitated. Sodium chloride was demonstrated to notably enhance the recovery of the residual proteins, especially FTL in the supernatant at pH 4.4 and pH 4.0. However, when the concentration of NaCl was further increased to >1.0 M, the outcome significantly declined. Consequently, the presence of sodium chloride ranging from 0.5 to 1.0 M, cell lysate with pH between 4.0 and 4.4, and incubating at 70 or 75℃ for 10 min would yield best results.

**TABLE 1:** Summary of the outcomes of precipitation process optimization

pH/Tempe		Retained protein recovery (%)					rhFTL purity (%)				
NaCl (M)		0	0.5	1.0	1.5	2.0	0	0.5	1.0	1.5	2.0
4.0	70℃	1.2	20.4	15.0	12.2	6.3	31.7	82.9	87.1	76.5	73.0
4.4		3.9	22.5	19.5	13.7	7.2	29.0	83.4	86.2	80.8	64.4
4.8		9.5	21.5	16.4	14.1	8.6	58.9	81.0	81.3	77.4	71.7
4.0	75℃	0.4	20.4	14.4	11.5	5.5	35.7	85.1	88.6	75.2	69.6
4.4		0.7	23.4	21.5	14.2	7.0	35.4	85.0	89.5	86.4	72.7
4.8		8.2	29.4	23.3	18.4	13.2	34.0	76.2	69.8	65.5	53.6

*Note*: Precipitation buffer is 100 mM NaAc‐AcH.

The retained protein in clarified supernatant was measured by Bradford method, and the purity of rhFTL in SDS‐PAGE analysis was roughly calculated by densitometric scanning.

### 3.3 Purification of the rhFTL by Capto Butyl

**FIGURE 3:**
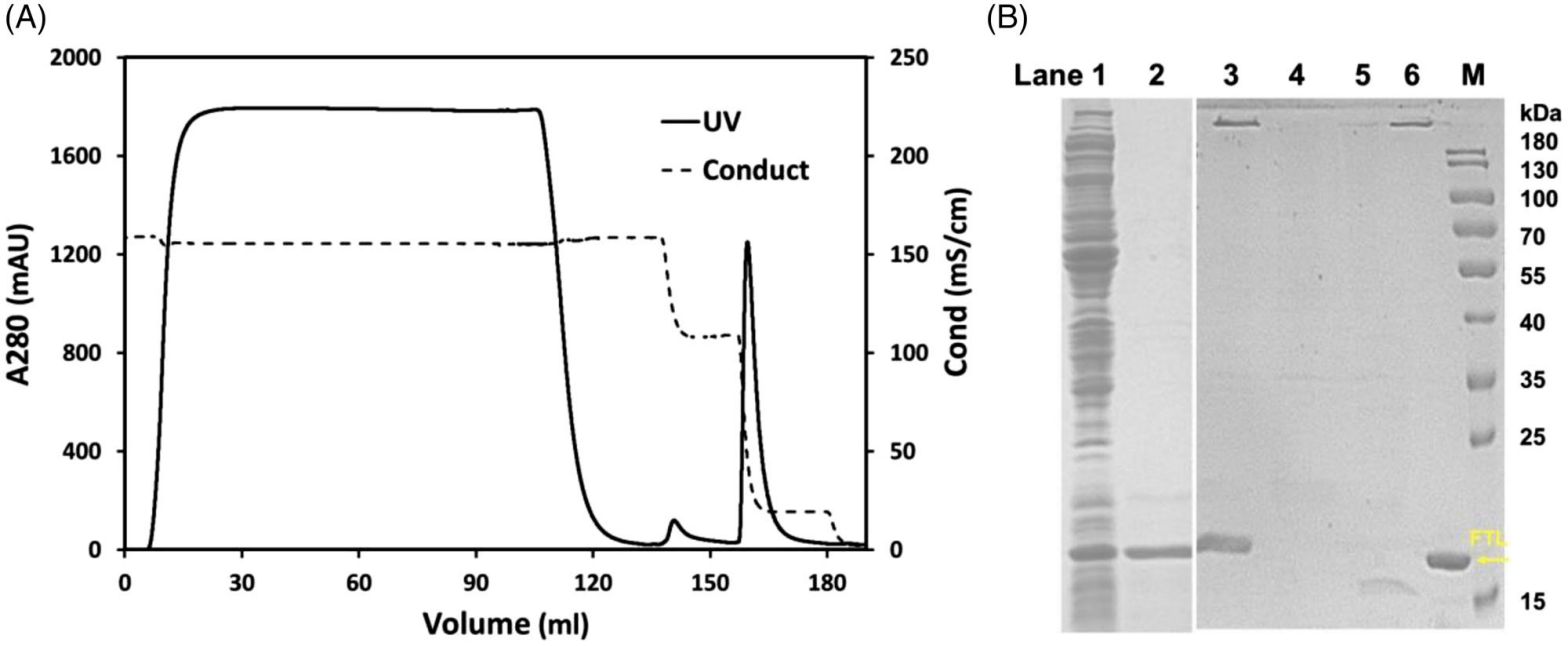
(A) Purification of rhFTL by hydrophobic interaction chromatography. (B) The intermediate samples analyzed by SDS‐PAGE. lane 1, crude lysate; lane 2, clarified supernatant; lane 3, loaded sample; lane 4, flow through sample; lane 5, 30% buffer B washed sample; lane 6, 90% buffer B eluted sample

**FIGURE 4:**
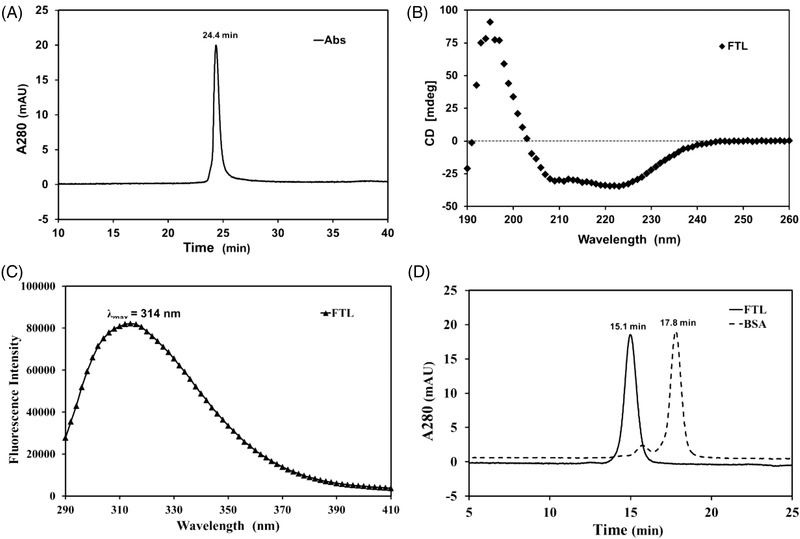
(A) RP‐HPLC analysis of the purity of rhFTL. Chromatographic conditions: 0–5 min, linear from 40% to 45% solution B; 5–35 min, linear from 45% to 65% solution B; flow rate: 0.5 mL/min; solution A, ddH_2_O supplemented with 0.1% TFA; solution B, CH_3_CN supplemented with 0.1% TFA; (B) Secondary structure determination of the rhFTL by far‐UV CD spectrum. Scanning wavelength range, 190 ∼ 260 nm; wavelength bandwidth, 1.0 nm; scanning speed, 1000 nm/min; response interval, 0.1 s; (C) Intrinsic fluorescence emission analysis of the rhFTL. The excitation wavelength was constant to 280 nm, and the intrinsic fluorescence emission signals were recorded from 290 to 410 nm; (D) The assembly feature study of the resulted rhFTL by HPLC‐SEC. The flow rate was constant to 0.5 mL/min and BSA was used as a size marker

The precipitated supernatant was loaded onto the Capto Butyl column with a binding capacity of approximately 12 mg total protein per 1.0 mL resin. The diluted supernatant (100 mL) with a concentration of approximately 0.62 mg/mL was loaded with no obvious proteins detected in the flow‐through sample. The chromatographic spectrum is shown in Figure 3A and the outcomes are listed in Table 2. rhFTL was eluted by 90% buffer B with a recovery of about 90% during this step. The whole recovery of rhFTL from the crude lysate supernatant was approximately 80%. SDS‐PAGE (Figure 3B) demonstrated that the purity of rhFTL was approximately 95.0% which was roughly calculated by densitometric scanning. Furthermore, RP‐HPLC analysis demonstrated that rhFTL was eluted at 24.4 min, and the purity was above 98.0% (Figure 4A). The molecular mass determined by ESI‐MS analysis showed that the purified rhFTL had a molecular weight of 19878.0 Da which coincides with the theoretical value (Figure S1).

**TABLE 2:** Purification of the rhFTL by hydrophobic interaction chromatography (HIC)

**Step**	**rhFTL (mg)**	**Purity (%)**	**Recovery (%)**
**Crude lysate**	∼70	∼25^a^	100
**Precipitation process**	62	∼92^a^	88.6
**HIC**	56	98.4^b^	90.3
		Whole recovery	∼80.0

^a^ The purity was calculated by densitometric scanning.

^b^ RP‐HPLC.

### 3.4 Structural characterization of rhFTL

#### 3.4.1 Secondary structure

Far‐UV CD spectra from 260 to 190 nm was measured and the result (Figure 4B) indicated that the second structure of the purified rhFTL exhibited in the α‐helix form (49.7%) and also contained small parts of the β‐strand form (6.1%), as calculated by the K2D3 tool [20], and was roughly similar to the previously disclosed structural features [6].

#### 3.4.2 Intrinsic fluorescence emission

To probe the advanced structures of the purified rhFTL, intrinsic fluorescence emission spectra were obtained and the results are shown in Figure 4C. The maximum fluorescence emission wavelength of rhFTL was approximately at 314 nm, which is substantially lower than that of free tryptophan (348 nm), implying that the aromatic amino acids were nearly wrapped inside and the purified rhFTL exhibited an ordered and compact structure.

#### 3.4.3 Assembly feature study

To verify the assembly features of rhFTL derived from *E. coli*, rhFTL was subjected to size‐exclusion chromatography. The main peak was located at approximately 15.1 min, while the BSA (67 kDa) which was used as a reference, was observed at around 17.8 min (Figure 4D). The main peak of rhFTL was at a 2.7 shift prior to that of BSA, indicating that the apparent molecular size of rhFTL was extremely large compared to that of BSA. The results demonstrated that rhFTL fairly exists in a multi‐subunit form.

#### 3.4.4 Particle size measurement

**FIGURE 5:**
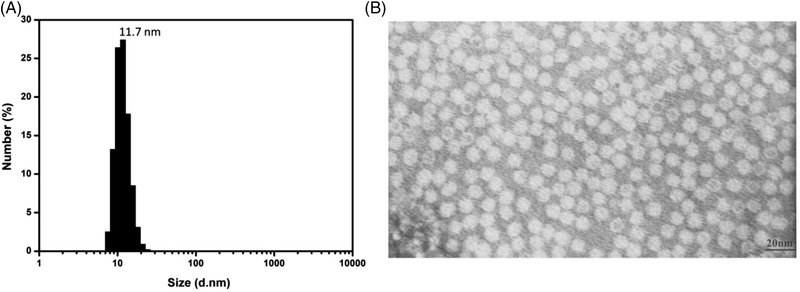
(A) Determination of the rhFTL particle size by DLS. The particle diameter of rhFTL represented an average size based on the mean number (%); (B) Observation of the morphological structure of rhFTL by TEM. The picture was shown at 150,000× magnification

DLS was subsequently conducted to probe the size of the obtained rhFTL and the results showed an extremely narrow particle size distribution. The median size was about 11.7 nm which is slightly smaller than that of the natural human ferritin (Figure 5A). This could be because the natural human ferritin contains multiple FTH (approximately 21 kDa) subunits, which have a slightly higher molecular weight than that of the FLC (approximately 19 kDa).

#### 3.4.5 Morphological structure

The specific morphological structure of rhFTL was observed using TEM and the results clearly demonstrated that rhFTL exhibits a spherical and highly uniform profile. The apparent molecular size was approximately 12 nm, according to the inherent marker (Figure 5B).

### 3.5 Thermal stress study

**FIGURE 6:**
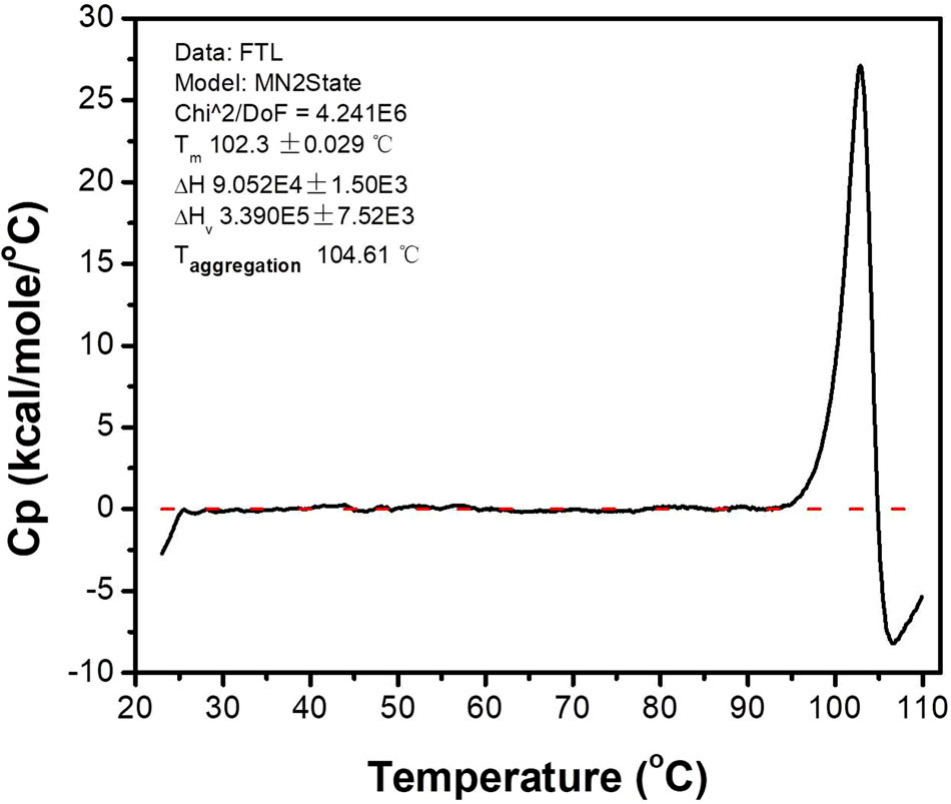
Measurement of the thermal stress response of the resulted rhFTL by DSC. The heating rate was 60℃/h and the scanning temperature was operated range from 20 to 110℃

DSC analysis was conducted to investigate the thermal stress response profile of rhFTL. The response curve demonstrated two key temperature points (*T*_m1_ = 102.3°C and *T*_m2_ = 104.6°C) (Figure 6). The onset temperature of the structural change was as high as 95°C. *T*_m1_ is approximately 102.3℃, indicating that the nanoparticle structure of rhFTL is extremely stable.

## 4 DISCUSSION

The use of *E. coli* to produce recombinant proteins always suffers from risking the formation of IBs. Moreover, the purification procedure also inevitably encounters complicated downstream processes due to the presence of a substantial amount of unwanted host (*E. coli*) proteins [21].Unlike FTH, it was previously reported that rhFTL expressed in *E. coli* tends to form IBs due to its relatively higher hydrophobicity than that of heavy chain [14, 15]. Although some efforts, such as co‐expression with many kinds of molecular chaperones facilitating FTL to fold into the correct structure and improving the ratio of soluble form were attempted, the outcomes remain unsatisfactory because of the extremely low yield [15]. Generally, the hydrophobicity of the protein itself, kinetic competition between aggregation and folding, and interactions with in vivo folding chaperones are the three most critical factors contributing to the formation of IBs in *E. coli* [22]. Thus, to retard the expression rate of rhFTL and change the in vivo folding environment in bacteria, we constructed a prokaryotic expression vector (rhFTL‐pBV220) using a pBV220 plasmid that contains a temperature‐sensitive pR/pL promoter. The pR/pL promoter in pBV220 plasmid demands higher temperature for induction, which can trigger host cell heat‐shock response, resulting in a certain degree of change in the in vivo folding circumstance [23]. As a result, the expression form of rhFTL was successfully and almost entirely converted to a soluble form and achieved a high yield of approximately 20%–30% of the total protein. Although the detailed mechanism of this conversion is not fully understood, two factors, including the retarded FTL expression rate and the potential folding chaperones resulting from the induction manner of pBV220 vector system or other heat‐shock response in host could reasonably be proposed to explain this scenario [24].

Ferritins derived from different species exhibit extraordinary thermal stability, and their spherical structure can temporarily withstand a temperature of 80℃ or more [25]. Thus, conventional strategies for extracting ferritins from complex crude materials always adopt a heating treatment mostly ranging from 70 to 80℃ for ∼10 min to remove most of unwanted proteins [26]. However, the subsequent process still necessitates multi‐step chromatography, such as anion exchange chromatography and size exclusion chromatography, to remove the residual impurities [14, 27]. FTL was reported to demonstrate a greater capability of resisting more severe conditions, including high temperature, strong denaturant, and extremely acidic pH than that of FTH because there is an extra salt bridge formed on the inner side of the FTL particle [28]. Therefore, to establish a simplified purification scheme, we developed a precipitation process based on the incubation temperature, pH, and ionic strength. The results showed that both 70 and 75℃ could remove most of the protein impurities under all the conditions, but 75℃ should be preferred because the delay of heat transfer needs to be considered during incubation. The impact of solution pH conditions ranging from 4.0 to 4.8 is of notable difference. At pH > 4.8, although the rhFTL was largely retained in the clarified supernatant, the impurities were not entirely precipitated and were difficult to remove in the subsequent process. In comparison, when the pH declined to 4.0, or even to 4.4, almost all unwanted proteins were substantially precipitated, however, the intended protein also co‐precipitated, leading to an extremely low recovery. Moderate concentrations of sodium chloride ranging from 0.5 to 1.0 M notably enhanced rhFTL recovery and did not significantly attenuate the removal of impurities at pH 4.4. Recent research also revealed that ionic strength can facilitate ferritin self‐assembly in vitro [29, 30]. Perhaps this benefit mainly ascribes to the formation of a salt‐bridge, which was reported to critically contribute to the physical stability of FTL [28]. In this work, further improving the sodium chloride concentration was not conducive to the recovery of rhFTL.

The retained rhFTL in the clarified supernatant was purified by one step of hydrophobic interaction chromatography (Capto Butyl). All types of the hydrophobic interaction chromatography resins, including Phenyl, Octyl, and Butyl‐S ligands, showed excellent recovery of rhFTL (data not shown), but Capto Butyl was preferred because the necessity of the ionic strength for rhFTL binding was moderate. Notably, the purification using Capto Butyl column used in this study contribute not mainly to the purity of protein, but to the excellent recovery of rhFTL. This was because it showed a fairly low separation between rhFTL and unwanted proteins, particularly when the impurities were not entirely removed during the precipitation process. CD and intrinsic fluorescence emission analysis indicated that the advanced structures of rhFTL were not disturbed by the harsh purification conditions. HPLC‐SEC and DLS results demonstrated that rhFTL exists in a fairly uniform multiple‐subunit structure with a particle diameter of approximately 12 nm. TEM indicated that the morphological structure of the purified protein was preserved. In addition, the thermal stress response profile showed that the onset temperature of the structure change was around 95℃, suggesting that the particle structure of rhFTL was extremely stable. *T*_m1_ was as high as 102.3℃, indicating that the spherical structure of rhFTL tended to be entirely unaffected when the crude lysate was provisionally treated at around 80℃ or even higher temperatures. Thus, it is reasonable to deduce that rhFTL could withstand with pH values ranging from 4.0 to 4.4 and temperature of 75℃ for 10 min.

## 5 CONCLUDING REMARKS

In summary, rhFTL was highly expressed and successfully converted to a soluble form, and thus it was successfully self‐assembled into a spherical structure in *E. coli* bacteria. The precipitation process using optimized incubation temperature, pH and ionic strengths was shown facile and efficient to remove those unwanted proteins. After purified by hydrophobic interaction chromatography, the production of rhFTL can be achieved with an excellent purity and a considerable recovery.

## CONFLICT OF INTEREST

The authors declare no conflicts of interest.

## Supporting information

Supporting Information

## Data Availability

The data that support the results of this study are available from the corresponding author upon reasonable request.
