# Supplementary material for: Conversion of recombinant human ferritin light chain inclusion bodies into uniform nanoparticles in Escherichia coli for facile production
Source: Eng Life Sci. 2022 Apr 23;22(6):453–63. doi: 10.1002/elsc.202100164 (PMC9162929; doi:10.1002/elsc.202100164)

Conversion of recombinant human ferritin light chain inclusion bodies into uniform nanoparticles in *Escherichia coli* for facile production

FIGURE 1 HPLC-ESI-MS analysis the detailed molecular mass of rhFTL. Chromatographic conditions: 0 ~ 20 min, linear from 5% to 90% solution B; 20 ~ 90 min, 90% solution B; flow rate, 0.2 ml/min; mobile phase A, 0.1% formic acid in ddH_2O; mobile phase B: 0.1% formic acid in CH_3CN

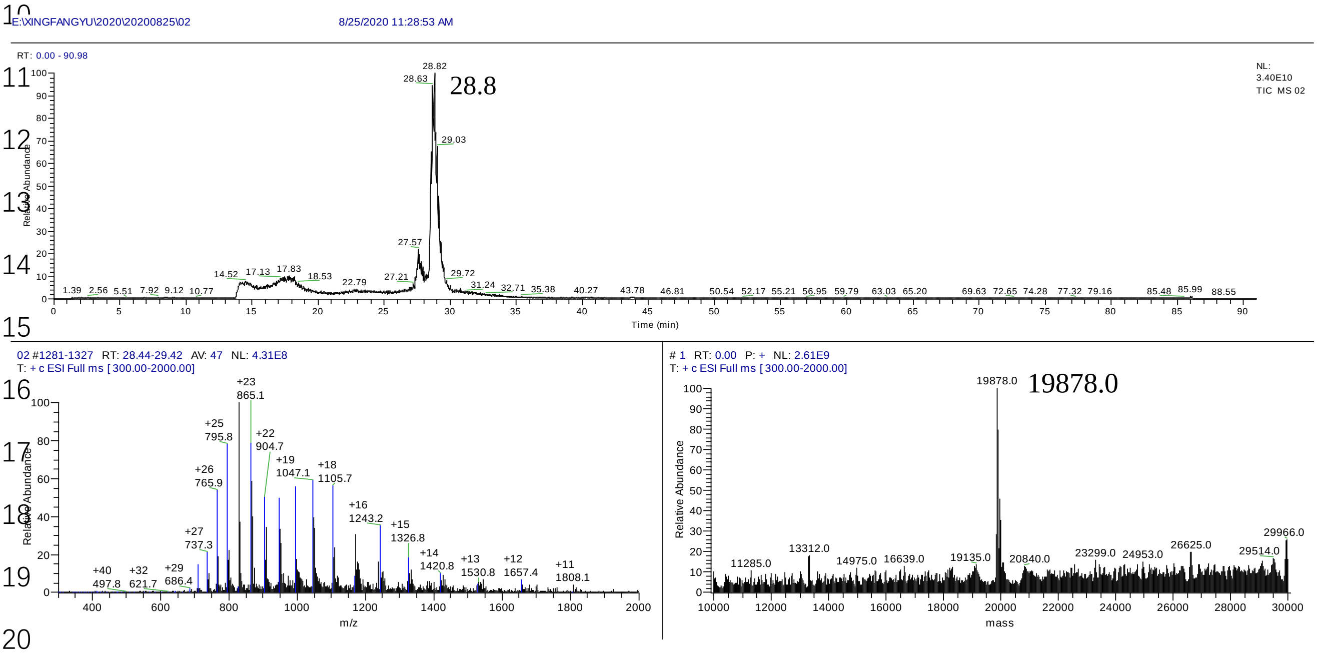

Supplement: Supplementary file 1 — Supporting Information [file ELSC-22-453-s001.pdf]
